# Bradycardia and syncope as sole manifestations of a cranial lesion: a case report

**DOI:** 10.1186/s13256-020-2345-8

**Published:** 2020-01-31

**Authors:** Dmitri Pchejetski, Mojiba Kenbaz, Heba Alshaker, Kiruparajan Jesudason

**Affiliations:** 1 James Paget University Hospital, Great Yarmouth, Norfolk, UK; 2 School of Medicine, University of East Anglia, Norwich, Norfolk UK; 3 2.53, Bob Champion Research & Educational Building, James Watson Road, Colney, Norwich, Norfolk NR4 7UQ UK; 4 Department of Pharmacology and Biomedical Sciences, Faculty of Pharmacy and Medical Sciences, University of Petra, Amman, Jordan

**Keywords:** Bradycardia, Syncope, Ectopic beats, Glioma, Brain lesion

## Abstract

**Background:**

Bradycardia and syncope are known sequelae of brain lesions. However, in the absence of neurological signs and symptoms, bradycardia and syncope are often investigated purely from the cardiovascular perspective and central nervous system-related causes may be easily overlooked during differential diagnosis.

**Case presentation:**

Here we report a case of a 69-year-old Caucasian man who presented to the emergency department after a fall. He had 1-year history of syncope and bradycardia with frequent ectopic beats shown on his electrocardiogram. He had no neurological symptoms. He was previously investigated as an out-patient and a diagnosis of idiopathic bradycardia with ventricular ectopic beats was made. On admission, cardiovascular investigations could not reveal the cause of his bradycardia. Computed tomography and magnetic resonance imaging scans of his head showed a localized mass in left basal ganglia consistent with infiltrating glioma.

**Conclusion:**

To the best of our knowledge this is the first case report demonstrating central nervous system-related bradycardia and syncope without other neurological symptoms. This case will serve as a useful reminder to general practitioners, accident and emergency doctors, and cardiologists.

## Background

Syncope is a clinical syndrome in which transient loss of consciousness (TLOC) is caused by a period of inadequate cerebral nutrient flow, most often the result of an abrupt drop of systemic blood pressure. It is a common presentation in primary care or in accident and emergency (A&E) settings. Causes range from the non-serious (such as vasovagal or orthostatic hypotension) to the potentially fatal (such as obstructive cardiac lesion). Broadly, causes of syncope can be classified as: metabolic, infectious, cardiac, and central nervous system (CNS) related. Various medications, such as beta blockers, may also cause bradycardia-induced syncope.

In the presence of bradycardia or abnormal heart rhythms, syncope is often investigated in the context of these changes and the most common causes are: cardiac arrhythmias, obstructive cardiac lesions, structural cardiopulmonary disease, sick sinus syndrome, Adams–Stokes syndrome, subclavian steal syndrome, or aortic dissection.

This case highlights the importance of considering other causes of syncope (such as metabolic or CNS related) even in the presence of cardiac findings. To the best of our knowledge, this is the first case report demonstrating CNS-related bradycardia and syncope without other neurological symptoms. This case will help general practitioners, A&E doctors, and cardiologists to formulate the differential diagnosis and request appropriate investigations.

## Case presentation

A 69-year-old Caucasian man came to A&E complaining of episodes of lightheadedness. He described a sensation beginning in his feet, spreading upwards, and then becoming lightheaded, hot, and clammy. Each episode lasted for a few minutes. The episodes occurred randomly in the past year, were not provoked by any stimuli, and there were no focalized neurological symptoms.

In general, he had felt unwell for the past year, feeling tired and weak, had reduced appetite, but no weight loss.

He had a past medical history of hypertension treated with ramipril. He was previously investigated for ischemic heart disease with exercise tolerance test that was negative for ischemic changes and 90% predicted heart rate was achieved. His echocardiogram showed good global systolic and diastolic function. He was previously investigated for syncope with a 7-day electrocardiogram (ECG) tape recording, which showed sinus rhythm with 12 episodes of bradycardia in 24 hours, the longest for 27 beats, lowest rate 23 beats per minute (bpm), ventricular ectopic beats, and pauses in cardiac activity < 2.5 seconds (at random times), and no ischemic changes. The pauses occurred at random times and were not related to any physical activity or time of day. This was not investigated further.

He does not smoke tobacco; he is a social drinker. He is a retired engineer and lives with his wife in a house, completely independent in his daily activities.

On examination, his temperature was 36.9, blood pressure 170/100 mmHg, oxygen saturation 98% on room air, and respiratory rate 12. His heart rate was 54. CNS, peripheral nervous system, chest, heart, and abdominal examinations were normal. There was no chest pain.

Metabolic, infectious, cardiac, and CNS causes for syncope were considered for the differential diagnosis. His blood tests were normal (Table 1), venous blood test was normal (not shown), and lying and standing blood pressures were 170/100 and 160/100, respectively. His heart rate was 54 bpm without orthostatic changes. Urine analysis and chest X-ray were normal, ruling out significant infection.

**Table 1:** Blood analysis on admission

Test	Result	Reference range
C-reactive protein	< 1	0–10 mg/L
White cell count	9.1	4–10 × 10^9^/L
Hemoglobin	142	130–170 g/L
Platelets count	251	140–400 × 10^9^/L
Mean cell volume (MCV)	89.5	80–100 fL
INR	1.05	
APTT (activated partial thromboplastin time)	28	22–36 seconds
Fibrinogen	3.38	2–4 g/L
Sodium	137	135–145 mmol/L
Potassium	4.8	3.5–5 mmol/L
Urea	5.5	2.5–6.5 mmol/L
Creatinine	99	55–120 μmol/L
Bilirubin	26	0–22 μmol/L
Alkaline phosphatase	58	20–140 U/L
Alanine transaminase	33	10–49 U/L
Albumin	37	32–48 g/L
Adjusted calcium	2.4	2.20–2.60 mmol/L
Troponin I	6.60	0 to 34.2 ng/L
TSH	1.26	0.35–3.50 mU/L
Free T4	14.2	7.5–21.1 pmol/L
Glucose		3.2–6.0 mmol/L

*INR* international normalized ratio, *T4* thyroxine, *TSH* thyroid-stimulating hormone

**Fig. 1:**
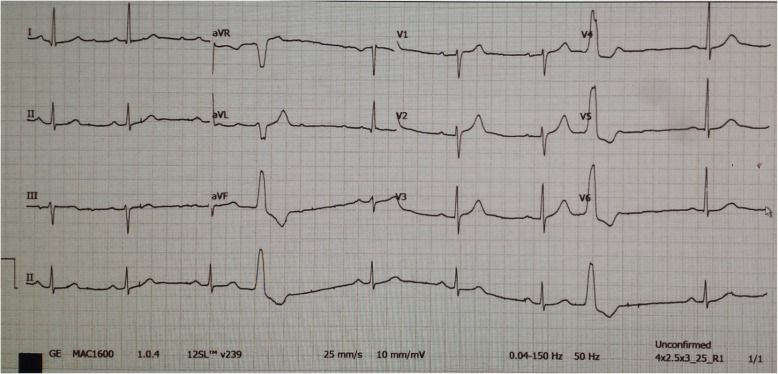
Electrocardiogram on admission

Heart block, ectopic beats, and arrhythmias were investigated by ECG, which showed sinus bradycardia of 54 bpm with occasional premature ventricular complexes and no features of acute ischemic event (Fig. 1).

Cranial causes for syncope include epilepsy, cerebrovascular accident (CVA), transient ischemic attack (TIA), space-occupying lesion, raised intracranial pressure, and trauma.

**Fig. 2:**
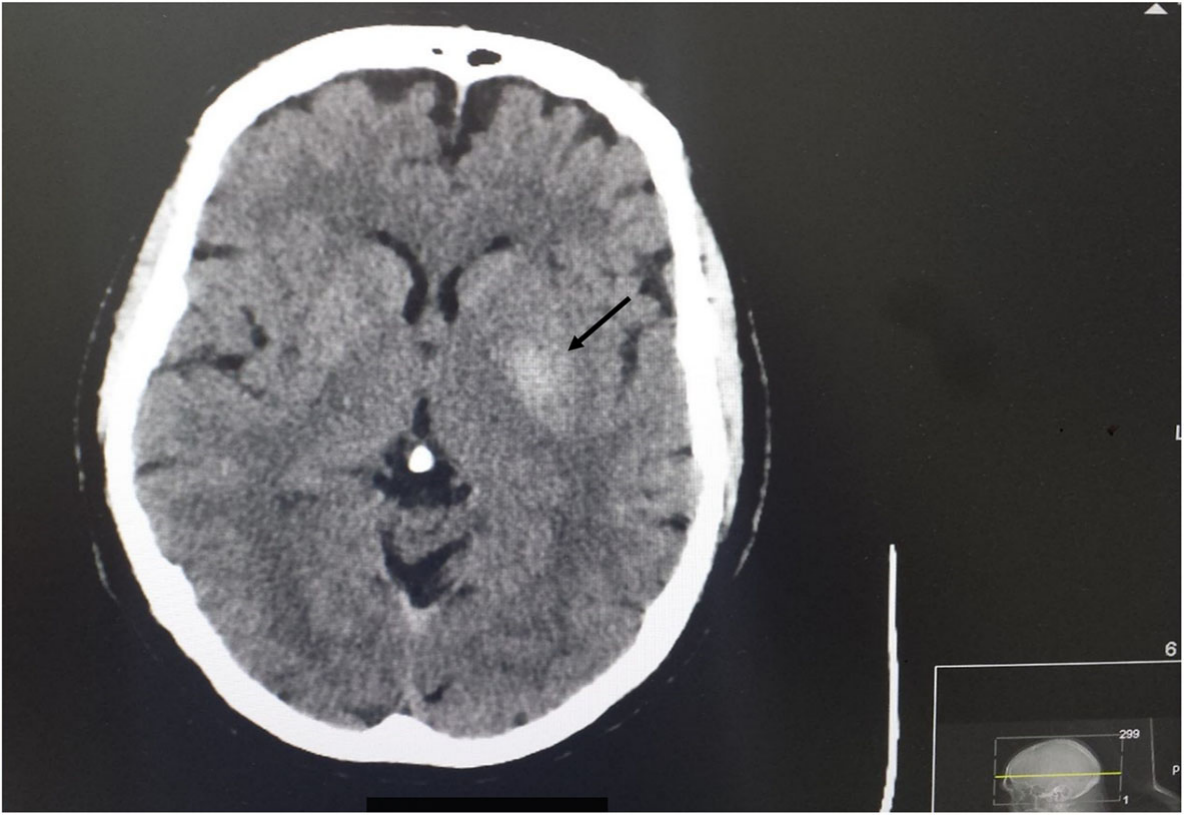
Computed tomography scan. Arrow: tumor

**Fig. 3:**
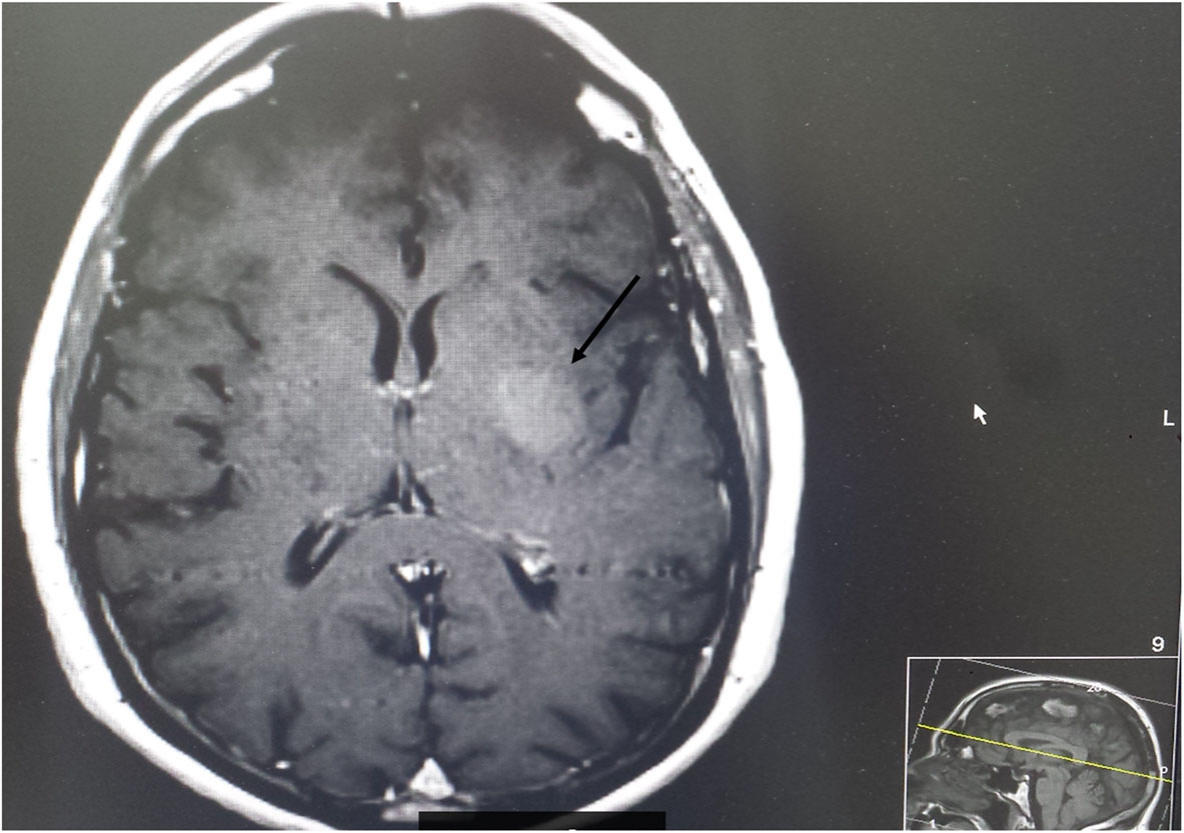
Magnetic resonance imaging scan. Arrow: tumor

A computed tomography (CT) head scan showed a hyperdense lesion 2.8 × 1.8 cm in left basal ganglia region, causing localized mass effect with minimal effacement of left sylvian fissure (Fig. 2). Our patient was admitted for further investigations. Based on CT findings, magnetic resonance imaging (MRI) was requested, which showed diffuse high signal and mild degree of enhancement in medial portion of left temporal lobe extending into globus pallidus and thalamus corresponding to CT appearance. These appearances were most consistent with infiltrating glioma (Fig. 3). A CT scan of his chest/abdomen/pelvis was then requested to assess for other cancer lesions. It demonstrated multinodular goiter, but otherwise no obvious primary or metastatic disease.

He had a primary tumor biopsy which showed anaplastic astrocytoma: isocitrate dehydrogenase (IDH) wild type, grade III, IDH r132h negative. He was discussed with a multidisciplinary team (MDT) and was treated with 8 mg dexamethasone and radiotherapy. His ECG improved showing 74 bpm sinus rhythm with first degree AV block. He is currently asymptomatic and has no syncopal episodes.

## Discussion

Here we report a case of a 69-year-old man who presented with 1-year history of syncope and bradycardia as the sole manifestations of an anaplastic astrocytoma. This is a rare presentation where a brain tumor causes no focal neurological symptoms and therefore can be missed during a diagnostic workup. In fact, this patient was previously investigated as an out-patient and a diagnosis of idiopathic bradycardia with ventricular ectopic beats was made. This case highlights the importance of considering other syncope causes (such as metabolic or CNS related) even in the presence of cardiac findings. To the best of our knowledge this is the first case report demonstrating CNS-related bradycardia and syncope without other neurological symptoms. This case will help general practitioners, A&E doctors, and cardiologists to formulate the differential diagnosis and request appropriate investigations.

Neurological causes of syncope are often overlooked in the presence of ECG changes and the absence of other neurological symptoms. The presence of primary cardiac abnormalities is often considered sufficient cause, while they may in fact be a mere consequence of neurological pathology. Several previously published papers have shown cardiac rhythm changes in patients with epilepsy [1–3], brain lesions [4, 5], or both [6]. In reported cases, brain tumor-associated syncope was also associated with other symptoms such as *jamais vu*, dizziness, and complex partial seizures with post-ictal confusion lasting for 30 minutes. These other symptoms pointing to a CNS cause allowed an easier differential diagnosis.

Brain cancers are rare. The reported incidence is 6 cases per 100,000. Approximately 60% of brain cancers are gliomas. The management of glioma is determined by a MDT and consists of: mostly surgery; radiotherapy; procarbazine, lomustine and vincristine chemotherapy; lomustine or temozolomide depending on grade and stage.

To the best of our knowledge, there are no reported cases demonstrating a CNS cause of bradycardia and syncope without other neurologic features. Both frontal [7] and temporal [6] lobe tumors were reported as causing bradycardia and syncope symptoms. Many different regions of the nervous system, including the brain stem, thalamus, hypothalamus, amygdala, and insular cortex are involved in cardiovascular control [8]. Pathology in these regions can give rise to various types of cardiac dysfunction. For instance, cortical stimulation studies in humans have shown depressor responses and bradycardia upon stimulation of the left insular cortex, whereas the converse applied for the right insular cortex [9]. A change in the heart rate was reported by the electrical stimulation of the cingulated gyrus and orbitofrontal cortex*.* It was hypothesized that these areas are interconnected to the central autonomic network [7, 10].

## Conclusions

In conclusion, this case highlights the importance of comprehensive differential diagnosis of syncope. As demonstrated in this case report, cerebral tumors can lead to cardiac arrhythmias and syncope without manifestations of “classical” CNS-related symptoms, such as focal neurology or seizures. In patients with “idiopathic” bradycardia or syncope, a brain tumor should be considered a differential diagnosis and a CT scan of the head or MRI of the brain should be requested. This case will help general practitioners, A&E doctors, and cardiologists to formulate the differential diagnosis and request appropriate investigations.
